# *Croton urucurana* Baill. Ameliorates Metabolic Associated Fatty Liver Disease in Rats

**DOI:** 10.3389/fphar.2022.886122

**Published:** 2022-05-20

**Authors:** Pablo Alvarez Auth, Gustavo Ratti da Silva, Eduarda Carolina Amaral, Victor Fajardo Bortoli, Mariana Inocencio Manzano, Lauro Mera de Souza, Evellyn Claudia Wietzikoski Lovato, João Tadeu Ribeiro-Paes, Arquimedes Gasparotto Junior, Francislaine Aparecida dos Reis Lívero

**Affiliations:** ^1^ Laboratory of Preclinical Research of Natural Products, Post-Graduate Program in Animal Science with Emphasis on Bioactive Products, Paranaense University, Umuarama, Brazil; ^2^ Laboratory of Preclinical Research of Natural Products, Post-Graduate Program in Medicinal Plants and Phytotherapeutics in Basic Attention, Paranaense University, Umuarama, Brazil; ^3^ Institute of Research Pelé Pequeno Príncipe, Pequeno Príncipe Faculty, Curitiba, Brazil; ^4^ Laboratory of Neurosciences, Post-Graduate Program in Medicinal Plants and Phytotherapeutics in Basic Attention, Paranaense University, Umuarama, Brazil; ^5^ Laboratory of Genetics and Cell Therapy, São Paulo State University, São Paulo, Brazil; ^6^ Laboratory of Cardiovascular Pharmacology, Faculty of Health Sciences, Federal University of Grande Dourados, Dourados, Brazil; ^7^ Laboratory of Preclinical Research of Natural Products, Post-Graduate Program in Medicinal Plants and Phytotherapeutics in Basic Attention, Post-Graduate in Animal Science with Emphasis on Bioactive Products, Paranaense University, Umuarama, Brazil

**Keywords:** dyslipidemia, euphorbiaceae, herbal medicine, hypertension, sangra-d’água, smoking

## Abstract

**Background:** Metabolic associated fatty liver disease (MAFLD) affects a quarter of the worldwide population, but no drug therapies have yet been developed. *Croton urucurana* Baill. (Euphorbiaceae) is a medicinal species, that is, widely distributed in Brazil. It is used in popular medicine to treat gastrointestinal, cardiovascular, and endocrine system diseases. However, its hepatoprotective and lipid-lowering effects have not yet been scientifically investigated.

**Aim of the study:** The present study investigated the effects of an extract of *C. urucurana* in a rat model of MAFLD that was associated with multiple risk factors, including hypertension, smoking, and dyslipidemia.

**Material and Methods:** The phytochemical composition of *C. urucurana* was evaluated by liquid chromatography-mass spectrometry. Spontaneously hypertensive rats received a 0.5% cholesterol-enriched diet and were exposed to cigarette smoke (9 cigarettes/day for 10 weeks). During the last 5 weeks, the animals were orally treated with vehicle (negative control [C-] group), *C. urucurana* extract (30, 100, and 300 mg/kg), or simvastatin + enalapril (two standard reference drugs that are commonly used to treat dyslipidemia and hypertension, respectively). One group of rats that were not exposed to these risk factors was also evaluated (basal group). Blood was collected for the analysis of cholesterol, triglyceride, alanine aminotransferase (ALT), and aspartate aminotransferase (AST) levels. The liver and feces were collected for lipid quantification. The liver was also processed for antioxidant and histopathological analysis.

**Results:** The main constituents of the *C. urucurana* extract were flavonoids, glycosides, and alkaloids. The model successfully induced MAFLD, reflected by increases in AST and ALT levels, and induced oxidative stress in the C- group. Treatment with the *C. urucurana* extract (300 mg/kg) and simvastatin + enalapril decreased plasma and hepatic lipid levels. In contrast to simvastatin + enalapril treatment, *C. urucurana* reduced AST and ALT levels. Massive lesions were observed in the liver in the C- group, which were reversed by treatment with the *C. urucurana* extract (300 mg/kg).

**Conclusion:** *C. urucurana* extract exerted promising hepatoprotective and lipid-lowering effects in a preclinical rat model of MAFLD.

## Introduction

Metabolic associated fatty liver disease (MAFLD) is characterized by the accumulation of fat in the liver, which can lead to several severe complications, including cirrhosis, liver failure, cellular hepatocarcinoma, and cardiovascular disorders (Eslam et al., 2020; Tilg and Effenberger, 2020). Approximately one billion people are affected by MAFLD worldwide, which has major clinical and economic impacts on society (Younossi et al., 2018).

Many risk factors and stressors have been shown to lead to severe MAFLD (Buzzetti et al., 2016). Such observations led to the hypothesis that genetic, viral, metabolic, and environmental stressors, or “hits,” accelerate progression from simple hepatic steatosis to more advanced stages of the disease. Examples of these hits include metabolic stressors, such as hyperglycemia, hypertriglyceridemia, and hypercholesterolemia, that are associated with cardiovascular diseases, especially systemic arterial hypertension. Environmental stressors that contribute to liver disease include nutritional factors, environmental pollutants, and smoking (Anty and Gual, 2019; Marchisello et al., 2019).

Epidemiological studies suggest that exposure to cigarette smoke accelerates the development of several liver diseases, including MAFLD, hepatitis C, and primary biliary cirrhosis, and increase the risk of developing cellular hepatocarcinoma (Jung et al., 2019; Takenaka et al., 2020). Equally important, obesity and dyslipidemia have been identified as independent risk factors for liver disease and can act synergistically in the development of MAFLD when associated with smoking (Kim et al., 2018). Thus, exposure to both metabolic stressors and cigarette smoke can worsen the intensity of liver disease, especially in obese individuals and patients with preexisting conditions, such as dyslipidemia and hypertension (Charatcharoenwitthaya et al., 2020; Johnston et al., 2020).

Although MAFLD is a common condition that affects a quarter of the population, there are no approved drug therapies. New treatments are under development, but therapeutic response rates have only been modest (Eslam et al., 2020). Thus, the search for new therapeutic agents is very important. The evolution of scientific research has provided greater insights into the ethnobotany, phytochemistry, and pharmacological effects of medicinal plants. Extensive ethnopharmacology studies are necessary to validate the popular use of plants, discover new bioactive components, and develop safe and effective herbal medicine and supplement formulations (Sen and Samanta, 2015).

A species, that is, widely used in Brazilian traditional medicine is *Croton urucurana* Baill. (Euphorbiaceae). This species, popularly known as “sangra-d’água” in Brazil, is a tree that can reach 7–14 m tall, with a 20 cm diameter trunk. Its leaves are heart-shaped and acquire a yellowish-red color when they are about to fall. When its trunk is cut or injured, it releases a sap that becomes resinous when it contacts air, acquiring a reddish color (Lorenzi and Matos, 2002; Costa et al., 2020). Its leaves, bark, and sap are popularly used as antiinflammatory, antiulcerogenic, analgesic, antidiarrheal, healing, and hepatoprotective agents (Gurgel et al., 2001; Rao et al., 2007; Cordeiro et al., 2016; Câ ndido-Bacani et al., 2017; Coelho et al., 2019). *Croton urucurana* is used in popular medicine to treat gastrointestinal, cardiovascular, and endocrine system diseases (Coelho et al., 2019). Among these diseases, dyslipidemia and hypertension are notable. MAFLD is an asymptomatic chronic disease, that is, linked to dyslipidemia. Excess lipids are a precipitating factor for the development of MAFLD, which generates hepatic disorders that are also prevented by the hepatoprotective action of *Croton urucurana* in its popular hepatoprotective use. The main constituents of *C. urucurana* are tannins, lignans, and alkaloids (Peres et al., 1997). Toxicological studies found that *C. urucurana* is potentially nontoxic, with an oral lethal dose 50 (LD50) > 5 g/kg in mice (Gurgel et al., 2005).

However, the efficacy of this medicinal species against liver diseases has not yet been pharmacologically investigated. Thus, the present study employed a preclinical model of MAFLD in rats that combined several risk factors, including dyslipidemia, hypertension, and tobacco smoking, and evaluated the hepatoprotective effects of a *C. urucurana* extract.

## Materials and Methods

### Drugs

Bovine serum albumin, 5,5′-dithiobis (2-nitrobenzoic acid) (DTNB), reduced glutathione (GSH), xylenol orange, K2HPO4, KH2PO4, 1 M Tris, ethylenediaminetetraacetic acid, Tris HCl (all from Sigma, St. Louis, MO, United States), pyrogallol, absolute ethanol, absolute methanol, ferrous ammonium sulfate, trichloroacetic acid, formaldehyde (all from Vetec, Rio de Janeiro, Brazil), and ultra-pure water from a Milli-Q system were used for eluent preparation.

### Plant Material

There are no local traditional medicinal plant books with species that are popularly used in the region of Grande Dourados, Mato Grosso do Sul, Brazil. Therefore, *Croton urucurana* Baill. was selected based on a broad ethnobotanical study that was conducted by our research group to identify botanical species that are used by healers in the region. Data were collected through semi-structured interviews and participant observations. We were able to obtain information on the preparation, use, and relative importance of this medicinal species. *C. urucurana* is popularly used as an infusion of its leaves or bark to treat cardiovascular and gastrointestinal disorders (Coelho et al., 2019).

### Extract Preparation

Leaves of *C. urucurana* were collected in May 2020 in Dourados, Mato Grosso do Sul, Brazil (“22°20.9299’south, 54°83.7713 west). A voucher specimen (no. 5536) was deposited at the Herbarium of the Federal University of Grande Dourados. The plant was dried in an oven and sprayed. The extract was prepared by infusion using the methodology of Barbosa et al. (2020). The pulverized material (100 g) was subjected to the extraction process by infusion with 1 L of boiling water. The resulting infusion was kept in an amber flask for 5 h. After filtration, the infusion was treated with 95% ethanol in a proportion of 1:3 (v/v) to precipitate proteins and polysaccharides, giving rise to a heterogeneous phase that was removed by filtration. The ethanol-soluble fraction was concentrated on a rotary evaporator and then lyophilized. The plant name was checked at http://www.theplantlist.org and found to be approved.

### Liquid Chromatography-Mass Spectrometry

The phytochemical composition of *C. urucurana* was evaluated by liquid chromatography-mass spectrometry (LC-MS) using a high-performance liquid chromatograph (Prominence LC 20A, Shimadzu) coupled to a Maxis 3G Q-Tof high-resolution mass spectrometer (Bruker Daltonics). Chromatography was performed on a C18 column (250 nm × 4.6 nm, 5 μM particle size; Phenomenex, Torrance, CA, United States), held at 40°C. The solvent was composed of ultra-pure water and acetonitrile (Lichrosolv-Merck) that contained 0.1% formic acid (for positive ion mode) or 0.1% ammonium formate (for negative ion mode). A linear gradient was developed at a flow rate of 1 ml/min, increasing acetonitrile from 5 to 80% in 25 min, then to 100% in 29 min, and then returning it to 5% in 30 min, followed by 5 min to rebalance the system before the next injection. The sample (2 mg/ml) was prepared in methanol-water. The injection volume was 20 μL. A photodiode array (200–400 nm) or high-resolution MS (m/z 100–1,500) was used for detection.

The high-resolution MS analyses were performed in positive ion mode and negative ion modes, with energies set at 500 V in the end-plate offset and 4.5 kV in the capillary. Nitrogen was used for sample desolvation. The dry gas flow was 8 L/min. The nebulizer pressure was 2 Bar. The source temperature was 250°C. Data-dependent analysis was performed to obtain fragmentation spectra by collision-induced dissociation-MS using argon as the collision gas, with a voltage ramp of 10–60 eV.

### Animals

The research model was developed in spontaneously hypertensive and Wistar Kyoto male rats, weighing 200–250 g, that were obtained from the central vivarium of the Federal University of Grande Dourados. The animals were housed in the vivarium of the Laboratory for Pre-Clinical Research of Natural Products at Paranaense University, with free access to food and water. They were housed under controlled environmental conditions (20°C ± 2°C temperature, 50 ± 10% relative humidity, 12 h/12 h light/dark cycle) with environmental enrichment. The total number of animals in the study was 48 (*n* = 8/group). The animals were weighed weekly on an analytical balance. The experimental protocol was approved by the Ethics Committee on the Use of Animals of Paranaense University (protocol no. 1000/2020). All national and international guidelines were followed. The reporting of animal investigations was performed and interpreted according to Animal Research Reporting of *in vivo* Experiments (ARRIVE) guidelines (Sert et al., 2020).

### Experimental Design

For 10 weeks, the animals received standard commercial food that was enriched with cholesterol *ad libitum* and were exposed to smoke from nine commercial cigarettes (0.8 mg nicotine, 10 mg tar, and 10 mg carbon monoxide) for 1 h daily, 5 days weekly, for 10 weeks, as proposed by Souza et al. (2020). For the induction of dyslipidemia, the animals received a commercial standard diet (Purina®) that was enriched with 0.5% cholesterol (150 g of standard feed for rodents, one egg yolk, and 13.5 ml of corn oil). All the ingredients were mixed with water, baked in an oven at 50°C for 36 h, and packed in vacuum bags as proposed by Souza et al. (2020). This preparation contains 225 mg cholesterol, 1.8 g saturated fat, 2.16 g monounsaturated fatty acids, and 0.72 g polyunsaturated fatty acids for every 150 g of standard feed for rodents. During the last 5 weeks of the study, the animals were treated orally, by gavage, with vehicle (0.1 ml of filtered water/100 g body weight; negative control [C-] group), the ethanol soluble fraction of *Croton urucurana* (30, 100, and 300 mg/kg), or simvastatin (SIM; 2.5 mg/kg) + enalapril (ENAL; 15 mg/kg), once daily. Normotensive, non-dyslipidemic, and non-smoke-exposed Wistar Kyoto rats (basal group) were treated with vehicle (filtered water; *n* = 8 The doses of the *C. urucurana* extract were defined according to its traditional use in Brazil (Coelho et al., 2019). The most-reported preparation is the use of 200 ml of pre-boiling water, that is, directly poured into an amount of crushed plant, that is, equivalent to a “closed hand” (∼2.5 g). The 30 mg/kg dose was calculated by dividing 2.5 g by the average weight of a human adult (70 kg). Thus, we used the 30 mg/kg dose, a 10-times higher dose (i.e., 300 mg/kg), and an intermediate dose on a logarithmic scale (100 mg/kg; Coelho et al., 2019). Electrocardiograms, heart rate, and blood pressure in these normotensive and hypertensive rats were described in a previous study by our group (Zago et al., 2021).

### Sample Collection

Blood samples were collected from the left carotid artery using heparinized syringes. Plasma was separated by centrifugation at 1,500 g × g for 10 min and stored at −80°C for the biochemical analyses. The rats were then euthanized by puncture of the diaphragm while under anesthesia, and the liver was removed. Samples were rapidly separated and frozen in liquid nitrogen to evaluate oxidative stress and perform biochemical analyses. Other organ samples were stored in 10% formalin solution for further histological analysis. Feces (representative of 2 days of feces accumulation) were collected directly from the animal cages on the last day of the experiment and stored at −20°C until processing.

### Plasma Biochemical Analysis

Plasma levels of alanine aminotransferase (ALT), aspartate aminotransferase (AST), cholesterol, and triglycerides were measured by the colorimetric enzymatic method in an automated analyzer (Quick Lab, São Paulo, Brazil).

### Measurement of Hepatic and Fecal Cholesterol and Triglycerides

Lyophilized liver and fecal samples underwent lipid extraction using the gravimetric method as described by Lívero et al. (2016). Liver and fecal samples were mixed with hexane as the solvent (1:10; feces:solvent) and heated at 80°C. After 12 h, the supernatant was transferred to a second flask and naturally evaporated. This procedure was repeated three times. The lipid content was then weighed and suspended in 1 ml of chloroform plus 2 ml of isopropanol to determine hepatic and fecal levels of triglycerides and cholesterol by the colorimetric enzymatic method in an automated analyzer. The percentage of lipids in the liver was calculated as the following: (lipids [%] = 100 × [final flask weight/initial flask weight]/0.1 g).

### Investigation of Hepatic Antioxidant System

To investigate the hepatic antioxidant system, liver samples were homogenized in a 1:10 dilution of potassium phosphate buffer (0.1 M, pH 6.5). Afterward, 100 µL was separated, suspended in 80 µL of trichloroacetic acid (12.5%), vortexed, and centrifuged at 6,000 rotations per minute (rpm; 15 min at 4°C) to analyze GSH levels (Sedlak and Lindsay, 1968). The remaining homogenate was centrifuged at 9,700 rpm for 20 min at 4°C to determine superoxide dismutase (SOD) activity and lipoperoxidation (LPO) levels according to Gao et al. (1998) and Jiang et al. (1992), respectively.

### Histopathological Analysis

A sample of the liver were fixed in buffered 10% formalin solution (distilled water, 35–40% formaldehyde, and monobasic and dibasic sodium phosphate), dehydrated with alcohol and xylene, embedded in paraffin, sectioned at 6 μM, and stained with hematoxylin/eosin. The other liver sample underwent saturation in sucrose (10, 20, and 30% sucrose solutions for 24 h at each concentration), stored in Tissue Tek (O.C.T. Sakura), rapidly frozen in liquid nitrogen, and sectioned (6 µM) for Nile Blue staining (Lívero et al., 2016). The slides were analyzed by optical microscopy (Leica DM 2500) to evaluate cellular alterations. Hepatic lesions were classified as the following: 0 (0%; absence of lesions), 0.5 (1–5%; minor lesions), 1 (6–33%; moderate lesions), 2 (34–66%; marked lesions), and 3 (67–100%; massive lesions) according to Souza et al. (2020).

### Statistical Analysis

The data were analyzed for homogeneity of variance and a normal distribution. Differences between means were determined by one-way analysis of variance (ANOVA) followed by the Newman-Keuls post hoc test or by the Kruskal-Wallis test followed by Newman–Keuls’s post hoc test. The level of significance was set at 95% (*p* < 0.05). The data are expressed as the mean ± standard error of the mean (SEM).

## Results

### Phytochemicals From *Croton urucurana*

The main constituents of the *C. urucurana* extract were identified as flavonoids and glycosides, along with alkaloids. High-resolution MS analysis was performed in positive and negative ion modes, but the positive polarity showed the best results—alkaloids were barely detected in negative ion mode. Thus, except as indicated, the results are presented in positive ion analysis, with neutral components from *C. urucurana* obtained as protonated ions [M^+^H]^+^.

**FIGURE 1:**
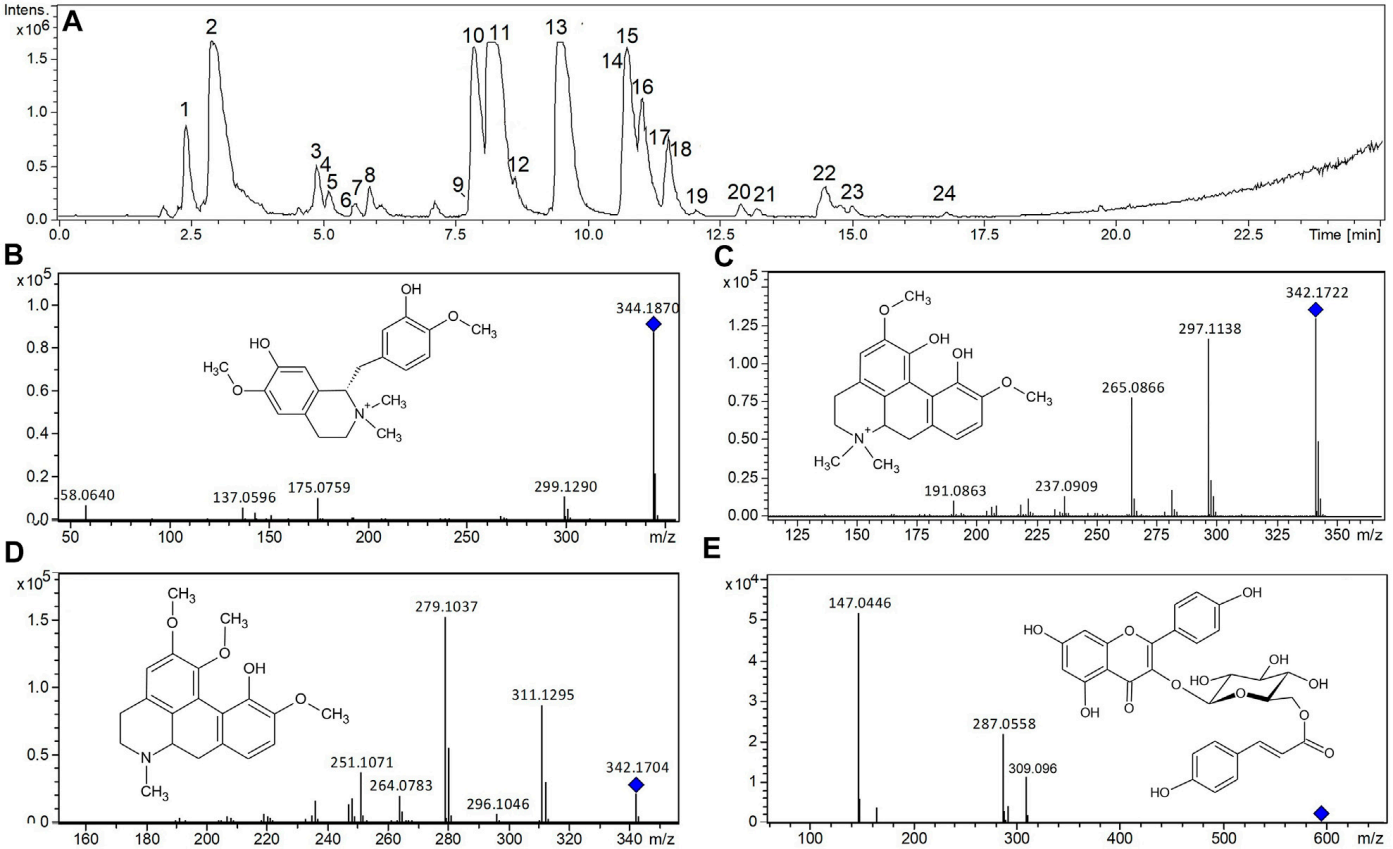
Chromatogram of the ethanolic soluble fraction that was obtained from the leaves of *Croton urucurana*. The compounds were detected in the positive ionization mass spectrometry **(A)**, numbered as elution profile order. **(B–D)** Tentative compound identification based on fragmentation profile mass spectra and proposed structures of the alkaloids tembetarine **(B)**, magnoflorine **(C)**, and isocorydine **(D)**. **(E)** The mass spectrum is consistent with the proposed structure of the flavonol-glycoside tiliroside.

Alkaloids are metabolic products, usually from amino acids. Therefore, they have a characteristically even m/z value because of the presence of a nitrogen element. Different peaks with even m/z values were observed in the *C. urucurana* extract. Although abundant on the chromatogram, peak one at m/z 266.124 and peak 2 at m/z 146.082 did not produce good fragments to allow their identification. Nevertheless, peak one had an m/z that was similar to the alkaloid anonaine (C_17_H_15_NO_2_), and peak 2 was similar to N-methyl-trans-4-hydroxy-L-proline that was extracted from *Myracrodruon urundeuva* (Aquino et al., 2019). Other low-abundance peaks (peaks 3–8) were observed with even m/z values, suggesting other alkaloid compounds (Figure 1A; Table 1). The most abundant peaks were also recognized as alkaloids, appearing at m/z 344.187 (peak 9), 342.172 (peak 11), 342.170 (peak 13), and 370.129 (peak 14). Cordeiro et al. (2016) described structures of some alkaloids that were obtained from the bark of *C. urucurana*, with the same m/z values as those observed herein. Peak 9 (m/z 344.187) gave fragments at m/z 299.129, 175.075 and, 143.049 and 137.059 (Figure 1B), with the fragment at m/z 299.129, with the neutral loss (NL) of 45.05 atomic mass units (a.m.u.) being consistent with the loss of a dimethylamine group. These fragments were the same as those found by Yan et al. (2013), identified as the aporphinic alkaloid tembetarine, which was consistent with our findings. Peak 11 at m/z 342.172 gave fragments at m/z 297.113 (NL of 45.05 a.m.u. from the loss of a dimethylamine group), 282.090, 265.086, and 237.090 (Figure 1C). Yan et al. (2013) described the structure that was found at the same m/z value, with similar fragments as those found herein, indicating that compound 11 is the alkaloid magnoflorine. A possible isomer also appeared at m/z 342.170 (peak 13). The fragmentation profiles of these compound were different from peak 11, with main fragments at m/z 311.129, 279.102, and 251.107 (Figure 1D). However, the NL of 31.04 a.m.u. that yielded the fragment at m/z 311.129 was consistent with the loss of a methylamine rather than dimethylamine as from compound 11. Singh et al. (2017) described the structure of an alkaloid, the aporphinic alkaloid isocorydine, in Mahonia leschenaultia with similar fragments. Peak 14 at m/z 370.129 was consistent with the structure of the alkaloid taspine, which was also described by Cordeiro et al. (2016).

**TABLE 1:** Analysis of phytochemicals identified in *Croton urucurana*, determined by LC-MS, with positive ion mass spectra results and tentative identification of each compound.

Peak	Rt (min)	MS^1^	MS^2^	Tentative identification	References
1	2.4	266.124	248.113	Anonaine	
2	2.9	146.082	-	Me-OH-proline	Alquino et al., 2019
3	4.9	294.155	230.139, 132.101	n.i.	
4	5.1	284.099	152.057	n.i.	
5	5.6	406.150	388.139, 342.132, 299.091 185.083	n.i.	
6	5.9	358.165	166.087, 120.081	n.i.	
7	6.1	328.139	310.129, 166.085	n.i.	
8	7.1	188.071	170.058, 146.060, 118.065	n.i.	
9	7.8	344.187	299.129, 175.075, 143.049 137.059	Tembetarine	Cordeiro et al., (2016), Yan et al., (2013)
10	7.9	579.147	289.070	di-(epi)catechin	Casao et al., (2020)
11	8.3	342.172	297.113, 282.090, 265.086, 237.090	Magnoflorine	Yan et al., (2013)
12	8.8	291.086	139.039	Catechin	Lopes Alves et al., (2020)
13	9.5	342.173	311.129, 279.103, 251.107	Isocorydine	Singh et al., (2017)
14	10.8	370.129	325.072	Taspine	Cordeiro et al., (2016)
15	10.8	611.164	303.052, 147.065	Rutin	Souza et al., (2016)
16	11.1	433.113	337.070, 313.071	(Iso)Vitexin	Lopes Alves et al., 2020, Prando et al., 2015
17	11.4	465.102	303.0503, 145.0498, 85.0275	Isoquercitrin	Lopes Alves et al., (2020)
18	11.5	595.166	449.108, 287.056	Kaempferol rutinoside	Souza et al., (2008b)
19	12.1	449.107	287.055	Kaempferol-hexoside	Souza et al., (2016)
20	12.9	197.117	179.106, 133.101	n.i.	
21	13.3	625.175	479.1160, 317.061, 129.054	Isorhamnetin di glycoside	Lopes Alves et al., (2020)
22	14.5	595.145	309.096, 287.055, 147.044	*trans*-tiliroside	Nascimento et al., (2017)
23	15	303.050	229.049, 153.017	Quercetin	Barboza et al., (2016)
24	16.7	287.055	258.052, 153.017, 121.027	kaempferol	Barboza et al., (2016)

n.i. = not identified.

Despite other studies that indicated the presence of various condensed tannins in *C. urucurana*, we observed a single peak at m/z 579.149 (peak 10) in the present extract, with a main fragment at m/z 289.070, which was characteristic of (epi)catechin-(epi)catechin, as previously described (Souza et al., 2008a; Casao et al., 2020). Peak 12 at m/z 291.0862 was identified as catechin, confirmed by comparison with an authentic standard (Zago et al., 2021).

Flavonol O-glycosides were also identified in the *C. urucurana* extract (peak 15 at m/z 611.163 and fragments at m/z 465.104 and 303.052). This compound was confirmed as rutin by comparison with an authentic standard. Flavone C-glycoside was also observed in the *C. urucurana* extract. This compound was observed at m/z 433.113 (peak 16), exhibiting the characteristic NL values of 90 and 120 a.m.u. that were observed mainly in negative ion mode, which were characteristic of vitexin and isovitexin (Prando et a., 2016). Peak 17 at m/z 465.105 was confirmed as isoquercitrin (quercetin-3-O-glucoside) by comparison with an authentic standard, as described by Lopes Alves et al., 2020, in *C. urucurana.* Peak 18 at m/z 595.165 with main fragments at m/z 449.107 and 287.055 was characteristic of kaempeferol diglycoside, such as kaempferol rutinoside or its isomers (Souza et al., 2008b). Compound 19 at m/z 449.107 with a main fragment at m/z 287.055 was identified as a kaempferol-O-hexoside, being glucosides and galactosides the most commonly found in plants (Souza et al., 2016).

Compound 21 at m/z 625.175 had main fragments at m/z 479.117 and 317.065. The sequential NL of 146.05 and 162.05 a.m.u. indicated the elimination of a deoxyhexosyl (e.g., rhamnose) residue, followed by the loss of an hexosyl (e.g., glucose or galactose) residue. The fragment at m/z 317.061 was consistent with methoxy-quercetin, in which we observed the loss of the radical CH_3_• in the negative fragment ions (Tirloni et al., 2018). Thus, compound 21 was tentatively identified as a methoxy-quercetin rutinoside (or isomer), similar to isorhamnetin-O-deoxyhexosyl-hexoside that was described by Lopes Alves et al., 2020.

Compound 22 at m/z 595.145 had a mass that was slightly less than the kaempferol rutinoside (m/z 595.165). This compound had two abundant fragments at m/z 287.055 (consistent with kaempferol) and m/z 147.044 (consistent with p-coumaroyl; Figure 1E). A compound known as trans-tiliroside has a p-coumaric acid, that is, attached to the glucose unit, that is, linked to the kaempferol (kaempferol 3-O-[6”-O-p-coumaroyl]-glucoside). In our analysis, a low-abundance fragment-ion appeared at m/z 309.096, indicating a p-coumaric ester that was linked to an hexosyl residue, as described in Croton cajucara (Nascimento et al., 2017). This strongly suggested that peak 22 was the trans-tiliroside compound. Compound 23 at m/z 300.050 was identified as quercetin, and compound 24 at m/z 287.055 was identified as kaempferol, based on comparisons with authentic standards.

### Effects of *Croton urucurana* Extract on Biochemical Profile

**FIGURE 2:**
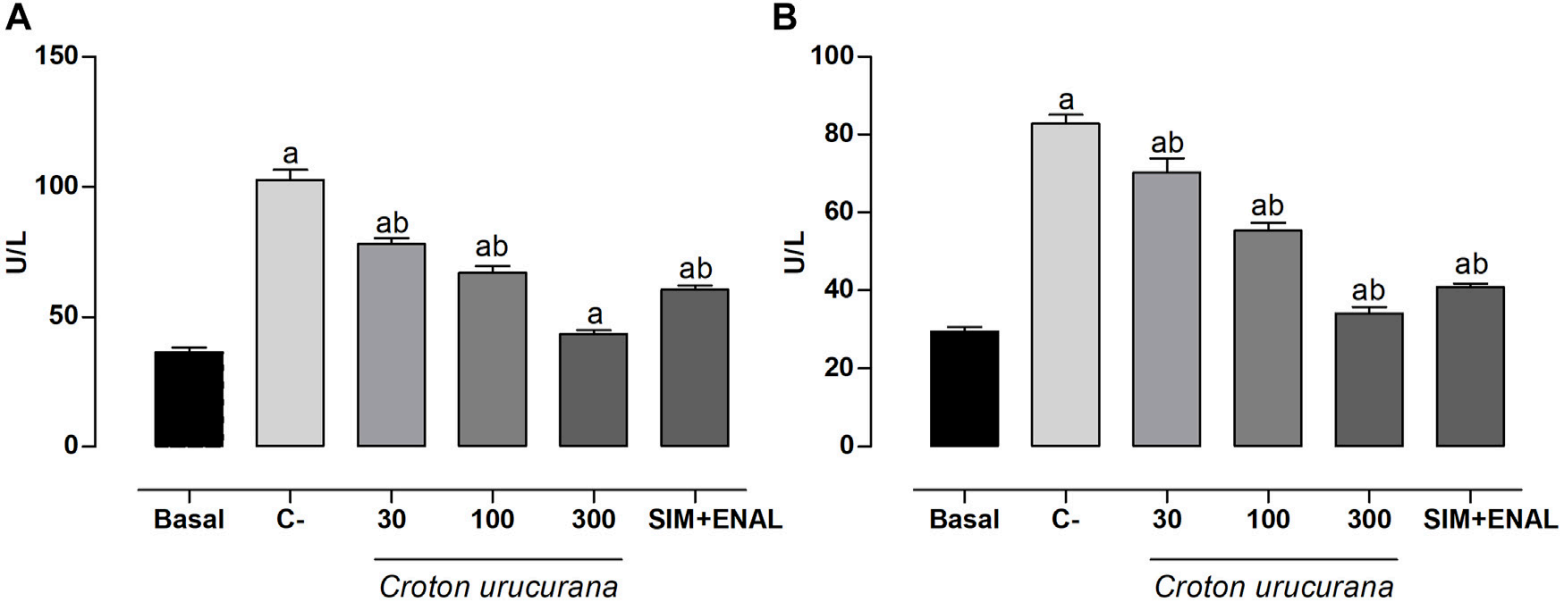
Levels of markers of hepatic damage. **(A,B)** Plasma levels of aspartate aminotransferase (AST; U/L) **(A)** and alanine aminotransferase (ALT; U/L) **(B)** in normotensive, non-dyslipidemic, and non-smoker rats (basal group) and hypertensive, dyslipidemic, and smoker spontaneously hypertensive rats that were treated with vehicle (negative control [C-]), *Croton urucurana* extract (30, 100, and 300 mg/kg), or simvastatin + enalapril (SIM + ENAL). The data are expressed as mean ± SEM. ap < 0.05, vs. basal; bp < 0.05, vs. C- group (one-way ANOVA followed by Newman-Keuls post hoc test).

Hypertension, dyslipidemia, and smoking increased ALT and AST levels by ∼180% compared with the basal group (29.50 ± 1.11 U/L and 36.53 ± 1.58 U/L, respectively). Treatment with 300 mg/kg *C. urucurana* extract completely reversed the increase in ALT and AST levels. Treatment with 30 and 100 mg/kg *C. urucurana* extract and SIM + ENAL partially reversed these changes (Figure 2).

### *Croton urucurana* Extract Exerted Lipid-Lowering Effects

Hypertension, dyslipidemia, and smoking increased plasma triglyceride and cholesterol levels by 203.90 and 429.76%, respectively, compared with the basal group. The risk factors also increased hepatic triglyceride and cholesterol levels compared with basal values. Treatment with 300 mg/kg *C. urucurana* extract and SIM + ENAL completely reversed these changes, whereas 100 and 300 mg/kg *C. urucurana* extract only partially reversed hepatic triglyceride and cholesterol levels. Finally, the C- group exhibited an increase in fecal triglyceride and cholesterol levels that were not reversed by any of the treatments (Table 2).

**TABLE 2:** Lipid profile in hypertensive, dyslipidemic, and smoker rats that were treated with vehicle, *Croton urucurana* extract, and simvastatin + enalapril.

	Basal	C-	*Croton urucurana*		
			30 mg/kg	100 mg/kg	300 mg/kg	SIM + ENAL
Liver weight (g)	14.5 ± 1.11	15.3 ± 0.80	14.1 ± 0.91	15.5 ± 0.79	14.1 ± 0.60	14.8 ± 0.70
Hepatic lipids (%)	15.80 ± 0.47	32.80 ± 1.58^a^	27.55 ± 1.43^ab^	21.98 ± 0.99^ab^	17.85 ± 0.51^b^	15.50 ± 0.47^b^
Plasma						
Cholesterol	33.30 ± 2.21	101.2 ± 4.03^a^	82.69 ± 1.89^ab^	66.69 ± 2.32^ab^	49.11 ± 1.78^b^	42.17 ± 2.65^b^
Triglycerides	43.17 ± 1.08	228.7 ± 10.71^a^	189.3 ± 4.29^ab^	135.9 ± 4.14^ab^	60.46 ± 2.65^b^	55.68 ± 3.58^b^
Hepatic						
Cholesterol	47.40 ± 2.80	208.8 ± 10.61^a^	177.8 ± 5.51^ab^	139.8 ± 4.96^ab^	73.27 ± 5.06^b^	52.73 ± 4.14^b^
Triglycerides	47.16 ± 2.67	117.5 ± 6.05^a^	95.42 ± 3.08^ab^	77.80 ± 1.53^ab^	48.74 ± 1.98^b^	44.20 ± 2.48^b^
Fecal						
Cholesterol	106.0 ± 4.17	196.0 ± 5.31^a^	186.8 ± 7.55^a^	185.1 ± 7.23^a^	186.0 ± 4.61^a^	189.7 ± 2.45^a^
Triglycerides	65.87 ± 5.95	118.9 ± 8.60^a^	112.0 ± 3.52^a^	109.4 ± 3.69^a^	105.8 ± 3.52^a^	99.07 ± 2.52^a^

a *p* < 0.05.

b *p* < 0.05.

Lipid profile in normotensive, non-dyslipidemic, and non-smoker rats (basal group) and hypertensive, dyslipidemic, and smoker spontaneously hypertensive rats that were treated with vehicle (negative control [C-]), *Croton urucurana* extract (30, 100, and 300 mg/kg), or simvastatin + enalapril (SIM + ENAL). The data are expressed as mean ± SEM. C- (one-way ANOVA, followed by Newman-Keuls post hoc test).

mg/dL, Cholesterol and triglycerides; C-, negative control group; SIM + ENAL, simvastatin + enalapril group.

### Effects of *Croton urucurana* on Hepatic Redox State

**FIGURE 3:**
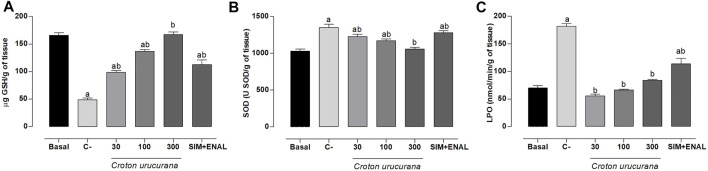
Antioxidant effects of *Croton urucurana*. **(A–C)** Hepatic levels of reduced glutathione **(A)**, lipoperoxidation **(B)**, and superoxide dismutase **(C)** in normotensive, non-dyslipidemic, and non-smoker rats (basal group) and hypertensive, dyslipidemic, and smoker spontaneously hypertensive rats that were treated with vehicle (negative control [C-]), *Croton urucurana* extract (30, 100, and 300 mg/kg), or simvastatin + enalapril (SIM + ENAL). The data are expressed as mean ± SEM. ap < 0.05, vs. basal; bp < 0.05, vs. C- (one-way ANOVA followed by Newman-Keuls post hoc test).

The combination of hypertension, dyslipidemia, and smoking induced significant hepatic oxidative stress (Figure 3). Decreases in hepatic GSH levels (48.83 ± 2.86 µg/g tissue) were observed compared with the basal group (165.9 ± 4.48 µg/g tissue). Increases in LPO and SOD levels were observed in the C- group compared with the basal group (69.95 ± 4.22 mmol/min/g tissue and 1,030.00 ± 27.18 U/g of tissue, respectively). Treatment with 300 mg/kg *C. urucurana* extract completely reversed these changes, whereas 30 and 100 mg/kg *C. urucurana* extract and SIM + ENAL only partially reversed these changes.

### Histological Evaluation of the Liver and Effects of *Croton urucurana*

**FIGURE 4:**
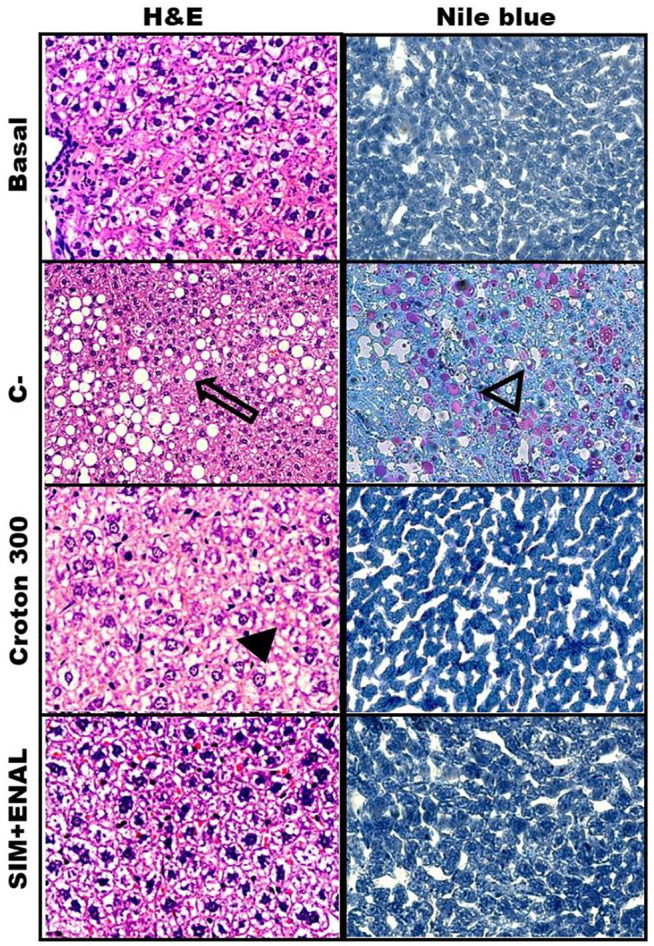
Histopathological hepatic analysis. Livers from normotensive, non-dyslipidemic, and non-smoker rats (basal group) and hypertensive, dyslipidemic, and smoker spontaneously hypertensive rats that were treated with vehicle (negative control [C-]), 30 mg/kg *Croton urucurana* extract (Croton 30), or simvastatin + enalapril (SIM + ENAL), stained with hematoxylin/eosin (A&E) or Nile Blue. The black arrow indicates steatosis. The open arrow indicates lipids droplets. The black arrowhead indicates tumefaction.

Liver histopathological alterations are shown in Figure 4. No alterations were observed in the basal group. Lesions in the C- group were classified as 3 (massive lesions). Treatment with 300 mg/kg *C. urucurana* extract exerted significant hepatoprotective effects (score = 0.5, minor lesions). Treatment with 30 and 100 mg/kg *C. urucurana* extract and SIM + ENAL exerted moderate hepatoprotective effects (score = 2, marked lesions).

## Discussion

**FIGURE 5:**
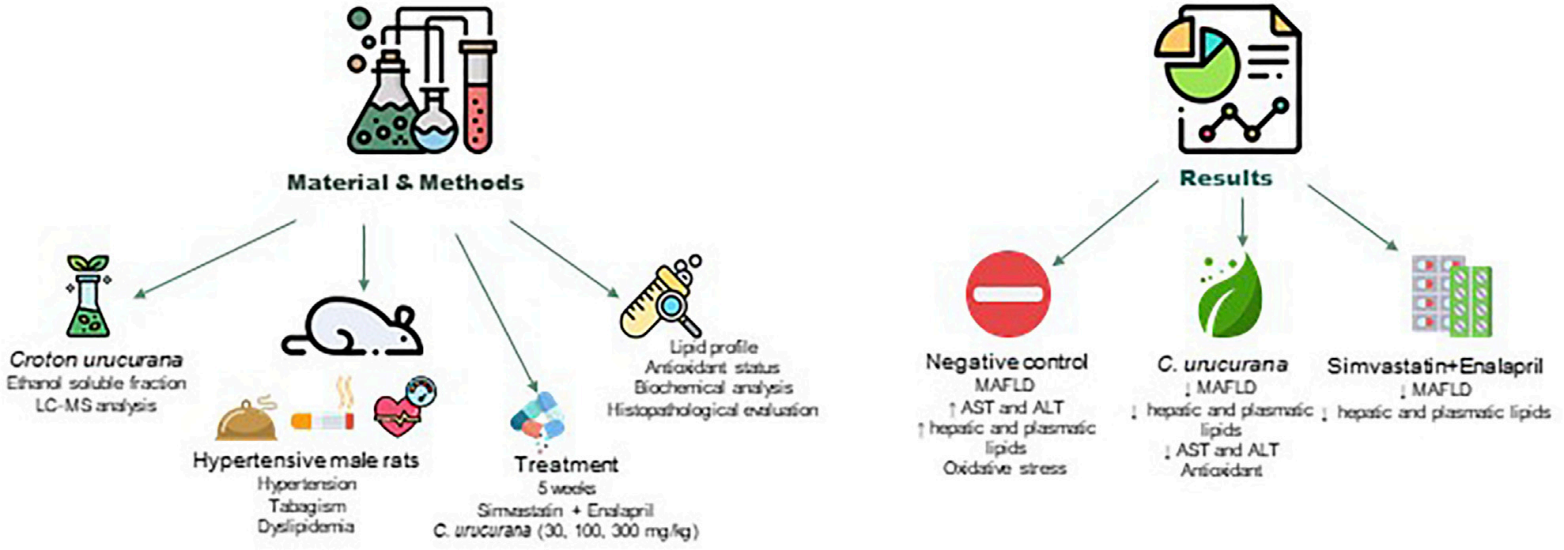
Summary of hepatoprotective effects of *Croton urucurana* using an experimental model that employed a combination of hypertension, dyslipidemia, and exposure to tobacco smoke.

The present study investigated the hepatoprotective effects of *Croton urucurana* using an experimental model that employed a combination of three important risk factors for MAFLD (i.e., hypertension, dyslipidemia, and exposure to tobacco smoke). Rats that were exposed to these risk factors for 10 weeks exhibited dyslipidemia, oxidative stress, and significant hepatic alterations. Prolonged daily treatment with an ethanol-soluble fraction from *Croton urucurana* leaves effectively reversed these changes (Figure 5).

Evidence indicates that the clinical importance of MAFLD is not limited to liver-related morbidity and mortality. It also extends to extrahepatic organs and several regulatory pathways, making MAFLD a multisystem disease (Armstrong et al., 2014; Byrne and Targher, 2015). However, despite the clinical relevance, most preclinical studies over the past few decades have addressed hypertension, smoking, dyslipidemia, and MAFLD in an isolated manner. Only recently, some models have been proposed that consider the synergistic contribution of these risk factors to the onset of MAFLD (Barbosa et al., 2020; Souza et al., 2020; Mendes et al., 2021). The present study supports further comprehensive research that seeks to reproduce the disease that occurs in humans in a more reliable way.

Among the risk factors for liver diseases, hypertension affects up to 50% of patients with MAFLD. Clinical evidence indicates that high blood pressure can promote disease onset and progression (Eslam et al., 2020; Kasper et al., 2021). The prevalence of hypertension is higher in individuals with MAFLD, indicating an important relationship to systemic inflammation, insulin resistance, and activation of the renin-angiotensin-aldosterone and sympathetic autonomic nervous systems (Ryoo et al., 2014; Lorbeer et al., 2017; Zhao et al., 2020; Zhou and Cen, 2020). With regard to tobacco smoking, the main mechanisms that are involved in triggering MAFLD include a reduction of adiponectin production (Iwashima et al., 2005; Kawamoto et al., 2010), increases in the production of erythropoietin and iron deposits in the liver (El-Zayadi et al., 2002), hepatic inflammation (Azzalini et al., 2010), oxidative stress (Muriel, 2009), and the activation of lipid synthesis (Yuan et al., 2009). In the present study, prolonged treatment with *C. urucurana* extract exerted actions on many of these targets, including lipid-lowering, antioxidant, and hepatoprotective effects, corroborating other preclinical studies that indicated that these actions are key to controlling and reversing MAFLD (Lívero et al., 2014, 2016; Barbosa et al., 2020; Souza et al., 2020).

Pharmacological studies of the development of new treatments for MAFLD have grown substantially in the past decade, but there are currently no approved drugs for its management (Thanapirom and Tsochatzis, 2019). The difficulty in developing such drugs for MAFLD can be attributed to its intrinsic and peculiar pathogenesis that involves several pathways and risk factors. Thus, therapies should control all events that lead to the development of steatosis and prevent cellular stress, inflammation, fibrosis, and cirrhosis (Stefan et al., 2019; Elhence et al., 2020).

In the present study, provide *in vivo* evidence for the acclaimed local use of *C. urucurana*. Studies indicated that *C. urucurana* is popularly used to treat diseases that affect the digestive system. The antiulcerogenic and antidiarrheal activity of this species has been pharmacologically evaluated (Miller et al., 2000; Gurgel et al., 2001; Rao et al., 2007; Barbieri et al., 2014; Cordeiro et al., 2016; Coelho et al., 2019). However, the hepatoprotective effects of *C. urucurana* have not yet been scientifically investigated. In the present study, classic dose-dependent effects were observed, and the hepatoprotective effects of *C. urucurana* extract were similar to the effects of standard drugs that are used to treat dyslipidemia (simvastatin) and hypertension (enalapril).

An important effect of the *C. urucurana* extract in rats was the reduction of lipids. This effect was statistically similar to SIM + ENAL treatment. Interestingly, we observed reductions of plasma and liver cholesterol and triglyceride levels but not fecal lipid levels. Thus, the mechanism of action of *C. urucurana* extract involves a reduction of plasma lipid levels but not a decrease in the intestinal absorption of cholesterol in the small intestine through the protein channel Niemann-Pick C1-like one protein (NPC1L1), which occurs with the lipid-lowering drug ezetimibe (Toyoda et al., 2019). The lipid reduction that was promoted by treatment with *C. urucurana* extract was similar to treatment with simvastatin. We hypothesize that the mechanism of action of this plant involves inhibition of the enzyme 3-hydroxy-3-methylglutaryl-CoA reductase. This lipid-lowering action of *C. urucurana* is important because the development of MAFLD requires an increase in hepatic uptake, *de novo* lipogenesis, and a decrease in fatty acid oxidation. This imbalance induces oxidative stress that promotes cell damage and disease progression (Ipsen et al., 2018).

The overall effects of *C. urucurana* were superior to the effects of SIM + ENAL. In addition to reducing levels of plasma and hepatic lipids, this plant extract also reversed oxidative stress that was induced by the three risk factors, which was partially observed with treatment with SIM + ENAL. These lipid-lowering and antioxidant effects of the plant can be attributed to the extract’s active metabolites. Several preclinical studies described these effects of flavonoids (Gou et al., 2020; Hu et al., 2020; Liu et al., 2021), glycosides (Bao et al., 2020; Kurek et al., 2021; Niu et al., 2021), and alkaloids (Cheng et al., 2020; Li et al., 2020; Ramos et al., 2021).

The superiority of *C. urucurana*’*s* effects was also observed with regard to liver damage markers (i.e., AST and ALT). Treatment with SIM + ENAL partially reversed the increases in the levels of these transaminases in the group of rats that was exposed to the three risk factors. This is an interesting beneficial effect. While exerting its lipid-lowering effects, *C. urucurana* did not cause liver damage, an adverse effect, that is, observed with drugs from the statin class (Meurer and Cohen, 2020). As expected, several animal models of MAFLD and alcoholic fatty liver disease have revealed increases in AST and ALT levels (Lívero et al., 2014, 2016; Barbosa et al., 2020; Souza et al., 2020; Mendes et al., 2021). The therapeutic effects of medicinal plants in reversing this increase in markers of liver damage are well described in the literature, especially the beneficial effects of flavonoids, glycosides, and alkaloids (Ling et al., 2020; Zhang et al., 2020; Han et al., 2021; Niu et al., 2021; Ore et al., 2021; Sahlan et al., 2021).

The present results add to the growing literature on MAFDL and may contribute to the further scientific validation of *C. urucurana* for the treatment of liver disease. One limitation of the present study was that we did not evaluate the molecular mechanisms that underlie the hepatoprotective effects of *C. urucurana* extract. We hypothesize that *C. urucurana* extract may also be beneficial against alcoholic and chronic liver diseases (Zhou et al., 2014; Lívero and Acco, 2015; Parola and Pinzani, 2019) because many pathways that are modulated by *C. urucurana* are involved in the pathogenesis of these diseases.

Plants remain an important source for the discovery of modern medicines. The findings of ethnobotanical studies continue to serve as a guide for the development of new drugs, with the assumption that many plants that have been historically used as traditional medicines likely have valid therapeutic potential (Atanasov et al., 2015; Buenz et al., 2018). Compared with synthetic drugs that are often evaluated randomly, medicinal plants with a long history of popular use are more likely to have effects on biological systems, thereby optimizing the time and cost associated with the research and development of new drugs, which are currently estimated to be 12 years and USD$291 million (David et al., 2015; Mohs and Greig, 2017; Jayasundara et al., 2019).

## Conclusion

*Croton urucurana* extract exerted promising hepatoprotective effects in a preclinical rat model of metabolic fatty liver disease. Our findings suggest that this species may be useful for treating patients with this disease, especially when associated with hypertension, smoking, and dyslipidemia. However, further studies are necessary to scientifically validate *C. urucurana* for the treatment of liver disease.

## Data Availability

The original contributions presented in the study are included in the article/Supplementary Material, further inquiries can be directed to the corresponding author.

## Abbreviations

a.m.u., atomic mass units; ANOVA, analysis of variance; ALT, alanine aminotransferase; AST, aspartate aminotransferase; *C. urucurana*, *Croton urucurana*; C-, negative control group; DTNB, 5,5′-dithiobis(2-nitrobenzoic acid); ENAL, enalapril; GSH, reduced glutathione; LC-MS, liquid chromatography-mass spectrometry; LD50, lethal dose 50; LPO, lipoperoxidation; MAFLD, metabolic associated fatty liver disease; NL, neutral loss; rpm, rotations per minute; SEM, standard error of the mean; SIM, simvastatin; SOD, superoxide dismutase.
